# Nitric oxide-elicited resistance to anti-glioblastoma photodynamic therapy

**DOI:** 10.20517/cdr.2020.25

**Published:** 2020-08-21

**Authors:** Albert W. Girotti, Jonathan M. Fahey, Witold Korytowski

**Affiliations:** 1 Department of Biochemistry, Medical College of Wisconsin, Milwaukee, WI 53226, USA.; 2 Department of Biophysics, Jagiellonian University, Krakow 31-008, Poland.

**Keywords:** Glioblastoma, photodynamic therapy, nitric oxide, inducible nitric oxide synthase, nitric oxide-mediated photodynamic therapy resistance, anti-nitric oxide adjuvants

## Abstract

Glioblastoma multiforme is a highly aggressive primary brain malignancy that resists most conventional chemoand radiotherapeutic interventions. Nitric oxide (NO), a short lived free radical molecule produced by inducible NO synthase (iNOS) in glioblastomas and other tumors, is known to play a key role in tumor persistence, progression, and chemo/radiotherapy resistance. Site-specific and minimally invasive photodynamic therapy (PDT), based on oxidative damage resulting from non-ionizing photoactivation of a sensitizing agent, is highly effective against glioblastoma, but resistance also exists in this case. Studies in the authors’ laboratory have shown that much of the latter is mediated by iNOS/NO. For example, when glioblastoma U87 or U251 cells sensitized in mitochondria with 5-aminolevulinic acid -induced protoporphyrin IX were exposed to a moderate dose of visible light, the observed apoptosis was strongly enhanced by an iNOS activity inhibitor or NO scavenger, indicating that iNOS/NO had increased cell resistance to photokilling. Moreover, cells that survived the photochallenge proliferated, migrated, and invaded more aggressively than controls, and these responses were also driven predominantly by iNOS/NO. Photostress-upregulated iNOS rather than basal enzyme was found to be responsible for all the negative effects described. Recognition of NO-mediated hyper-resistance/hyper-aggression in PDT-stressed glioblastoma has stimulated interest in how these responses can be prevented or at least minimized by pharmacologic adjuvants such as inhibitors of iNOS activity or transcription. Recent developments along these lines and their clinical potential for improving anti-glioblastoma PDT are discussed.

## INTRODUCTION

Malignant gliomas such as glioblastoma multiforme (GBM) are among the most aggressive and persistent of all known human tumors^[1–3]^. These malignancies are known to be highly resistant to most conventional interventions, including ionizing radiation and chemotherapy with drugs such as cisplatin and more recently introduced temozolomide^[4–6]^. Drug resistance can either be inherent or acquired during treatment^[5,6]^. One of the advantages of non-ionizing photodynamic therapy (PDT) is that it can often overcome or circumvent chemo- and radiotherapeutic resistance, yet various forms of treatment-induced resistance also exist for PDT^[7–9]^. One of these is based on nitric oxide (NO) produced by inducible nitric oxide synthase (iNOS/NOS2) in PDT-challenged tumor cells. This mode of resistance has been shown to be important not only for glioblastoma cells, but also several other human cancer lines, including breast, prostate, and melanoma^[10]^. Similar to chemotherapy, PDT involves a drug (photosensitizing agent), but resistance typically develops only after this agent is photoactivated to initiate photodynamic action. In this review, we discuss the following interrelated topics: (1) pro-survival role of constitutive NO in glioblastomas and other malignancies; (2) basic principles of PDT and the cytotoxic reactive oxygen species (ROS) produced; (3) mechanism of iNOS/NO induction by PDT; (4) hyper-resistance to PDT elicited by upregulated iNOS/NO; (5) NO-dependent hyper-aggressiveness of PDT-surviving cells; (6) tumor-supporting bystander effects of upregulated iNOS/NO; and (7) pharmacologic approaches for limiting the anti-PDT effects of NO.

## NITRIC OXIDE AND ITS PRO-SURVIVAL ROLE IN CANCER CELLS

**Figure 1.**
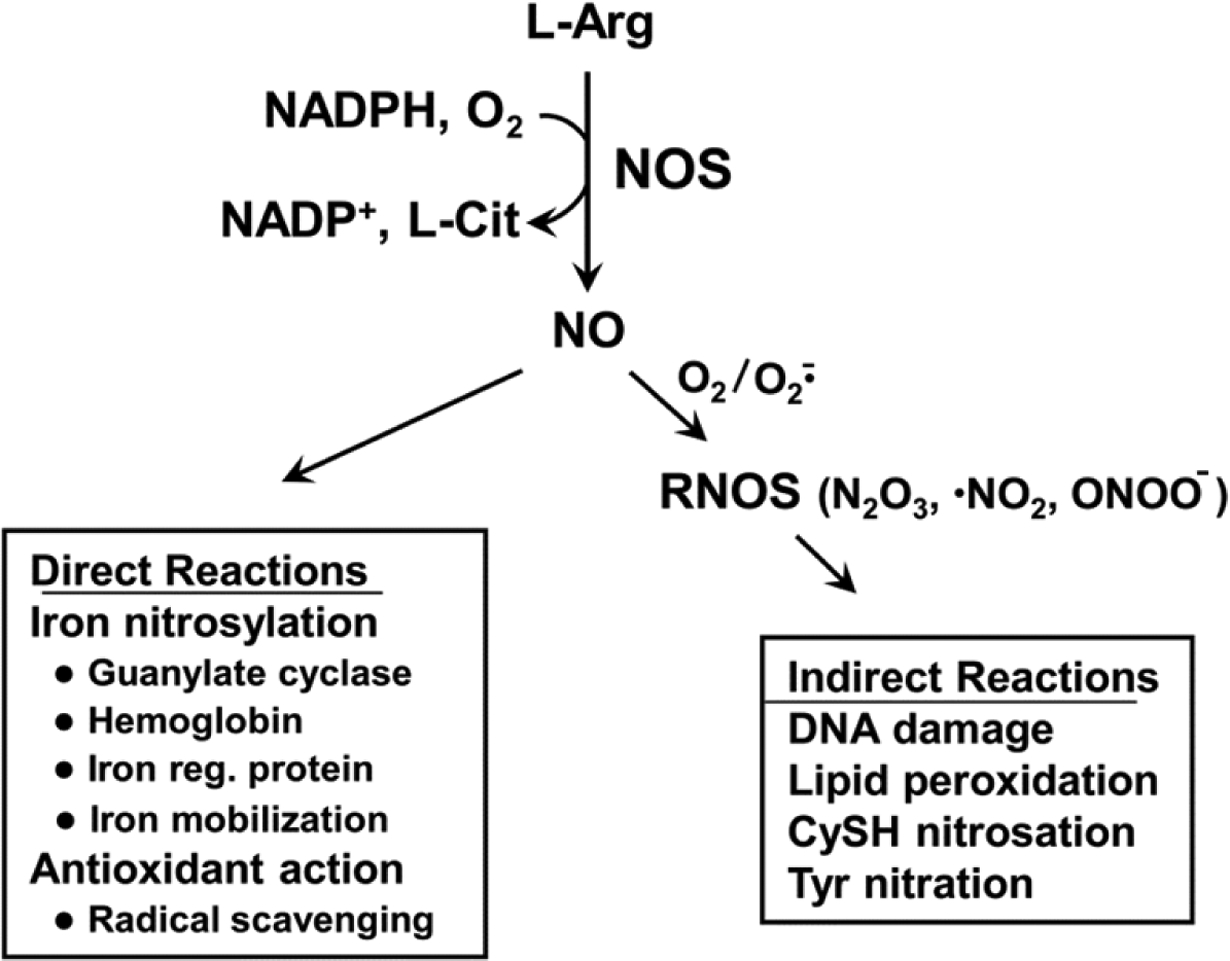
Generation of nitric oxide (NO) by nitric oxide synthase (NOS) enzymes. Examples of direct vs. indirect [reactive nitrogen oxide species (RNOS)-mediated] reactions of NO are shown

NO is a short-lived free radical molecule (τ < 2 s in H_2_O) generated by three different nitric oxide synthase enzymes: neuronal (nNOS/NOS1), inducible (iNOS/NOS2), and endothelial (eNOS/NOS3). All three isoforms are homodimers that catalyze the reaction of L-arginine with NADPH and O_2_ to give L-citrulline, NADP^+^, and NO [Figure 1]. Whereas nNOS and eNOS operate at constitutive levels, require Ca^2+^ for activity, and produce low levels of NO (< 0.1 μmol/L) in neurons and endothelial cells, iNOS does not require Ca^2+^, is induced by stress signals, e.g., in macrophages and neutrophils, and can generate much higher levels of NO (0.5–1 μmol/L)^[11]^. At low steady state levels, nNOS-derived NO is involved in neurotransmission, whereas eNOS-derived NO stimulates cyclic-GMP formation for vasodilation and regulation of blood pressure^[11]^. On the other hand, high level iNOS-derived NO produced by activated macrophages during an inflammatory response can be cytotoxic and potentially carcinogenic if it leads to DNA mutations^[11–14]^. NO may cause some of these effects by direct binding to iron in heme- or iron-sulfur proteins (nitrosylation), but it typically does so after conversion to more reactive species, e.g., oxidation to NO radical or reaction with superoxide radical (O_2_^−^) to give peroxynitrite (ONOO^−^) [Figure 1]. When generated under nitro-oxidative stress conditions, these species can cause oxidative damage to proteins, membrane lipids, and DNA^[12–14]^. It is now well recognized that many tumor cells, including glioblastoma cells, also express low levels of iNOS-derived NO and that this plays a key role in tumor persistence and progression as well as resistance to various therapeutic interventions^[15–17]^. At low-to-moderately high steady state levels (e.g., 10–400 nmol/L), tumor NO can activate signaling pathways by modifying/activating effector proteins such as soluble guanylyl cyclase (sGC), hypoxia-inducible factor-1α, extracellular signal-regulated kinases-1/2, protein kinase-B (Akt) via phosphoinositide-3-kinase (PI3K), and epidermal growth factor receptor (EGFR)^[18]^. These effectors respond to NO concentration in a graded way, e.g., sGC is activated by a very low level (5–10 nmol/L) and EGFR by a much higher level (~350 nmol/L)^[18]^. Modification typically occurs via S-nitrosation (SNO formation) at specialized cysteine residues whose sulfhydryl group pK values are much lower than normal^[12–14]^. As indicated in Figure 1, SNO formation does not occur via direct reaction with NO, but rather an oxidized form of NO such as nitrous anhydride^[19,20]^. SNO-mediated NO signaling can be attenuated as stress subsides, e.g., by thioredoxin- or glutaredoxincatalyzed SNO cleavage^[19,20]^. As pointed out above, highly aggressive tumor cells such as glioblastomas exploit endogenous iNOS/NO not only for hyper-proliferation and motility, but also resistance to ionizing radiation and chemotherapeutic agents such as cisplatin and docetaxel^[12–14]^. In pre-clinical trials, resistance to radiation or cisplatin could be partially overcome by pharmacologic inhibitors of iNOS enzymatic activity, consistent with NO involvement^[21,22]^. To date, however, none of these trials have dealt with cancer in general or brain cancer in particular, and none have employed anti-tumor PDT. Temozolimide (TMZ) has become one of the most widely used chemotherapeutics for glioblastoma; however, inherent or acquired resistance to this drug has limited its effectiveness^[5,6]^. Various approaches have been used in attempt to overcome this obstacle, but, thus far, it appears that the possibility of iNOS/NO-based resistance to TMZ has not been considered.

## PHOTODYNAMIC THERAPY FOR GLIOBLASTOMA AND OTHER SOLID TUMORS

**Figure 2.**
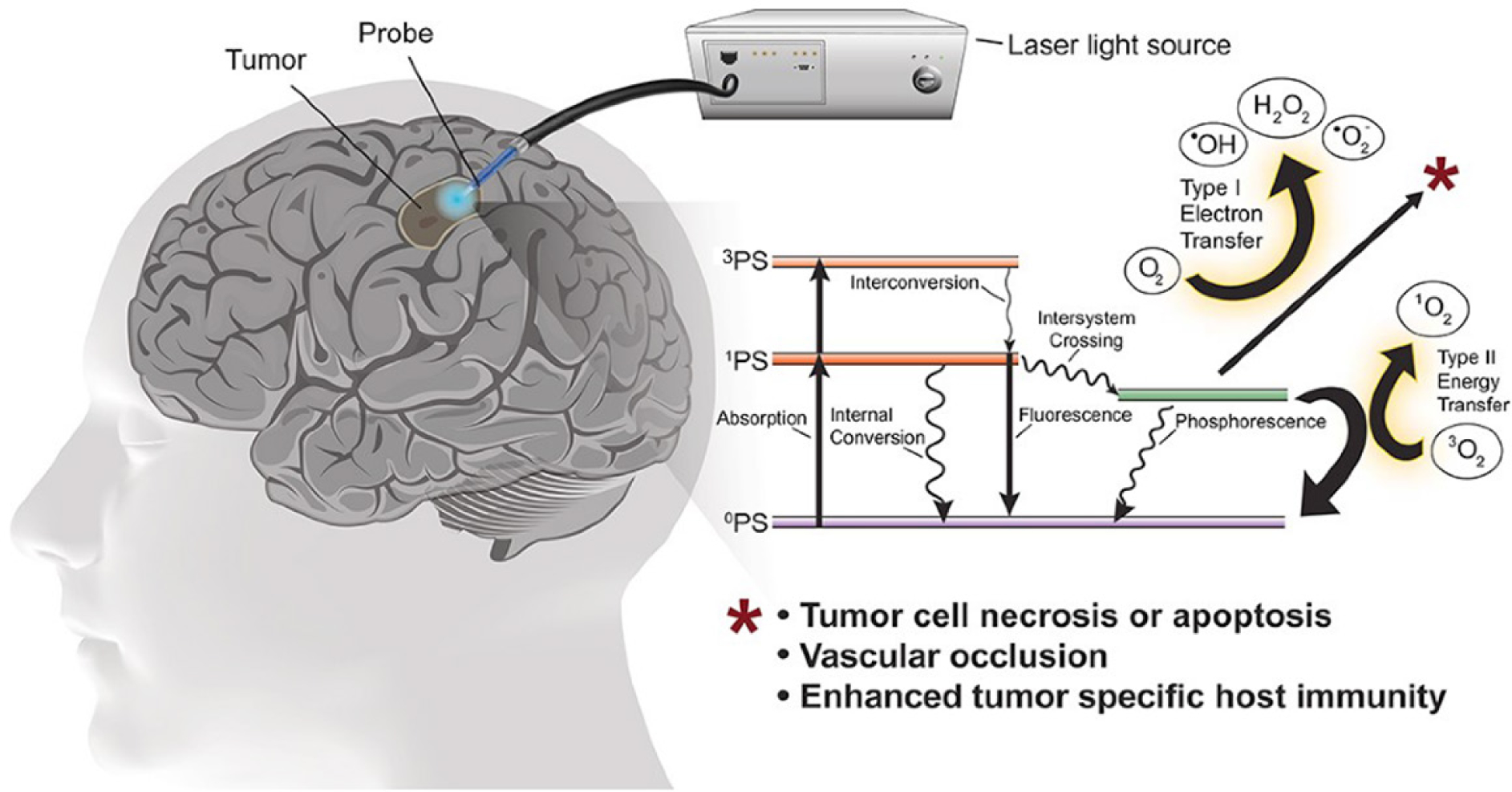
Illustration of interstitial photodynamic therapy for glioblastoma and other brain malignancies. A Jablonski diagram and reactive oxygen species-based mechanisms are depicted: Type I (free radical) vs. Type II (singlet oxygen)^[9]^

PDT was developed in the mid-1970s as a novel means for selectively eradicating a variety of malignant solid tumors via cytotoxic photochemistry^[23–25]^. This anti-tumor modality is minimally invasive and exerts little, if any, off-target cytotoxicity. Classical PDT consists of three operating components: (1) an administered photosensitizing agent (PS); (2) PS photoexcitation by non-ionizing radiation (typically in the far visible to near-infrared wavelength range); and (3) molecular oxygen^[24,25]^. All three components must be engaged concurrently to activate PDT, and light delivery via fiber optic networks makes clinical PDT highly selective for the intended tumor target. Most PS are innocuous until photoactivated and, unlike many chemotherapeutic agents, have few (if any) negative effects on normal tissues. A typical photodynamic reaction in PDT (Type II process) involves energy transfer from photoexcited triplet state PS to ground state O_2_, giving singlet molecular oxygen (^1^O_2_), a cytotoxic ROS [Figure 2]. For some PS, electron transfer to O_2_ may occur (Type I process), resulting in formation of free radical ROS. The latter, such as non-radical ^1^O_2_, can kill tumor cells by oxidizing vital molecules (proteins, lipids, and nucleic acids) and activating death signaling pathways^[25]^. Approximately 25 years ago, Photofrin®, a hematoporphyrin oligomer, became the first PS to receive FDA approval for clinical PDT on bladder cancer^[24]^. Since then, PDT with Photofrin® and other administered PS has been used for various other malignancies, including head and neck, breast, prostate, cervical, and brain (gliomas) cancers^[25]^. PDT has emerged as one of the most promising alternatives to chemo- and radiotherapy for treating glioblastoma and other brain malignancies [Figure 2]. A major reason for this is that PDT exhibits a very high level of tumor-specificity. It usually hits cytoplasmic targets, (e.g., mitochondria and endoplasmic reticulum), whereas chemotherapy (e.g., with TMZ) and radiotherapy typically target the nucleus (DNA).

Consequently, PDT can often circumvent any resistance that may exist/arise against these other therapies. Moreover, anti-tumor immunity is known to be enhanced by PDT^[26]^, and this is an additional advantage of this therapy.

In addition to pre-existing PS such as Photofrin®, which are applied as such, pro-sensitizers have been developed, one widely used type being 5-aminolevulinic acid (ALA)^[27–30]^. ALA enters cells via an amino acid transporter and is metabolized to the actual PS, protoporphyrin IX (PpIX), via the heme biosynthetic pathway. To accommodate pro-growth/expansion needs, this pathway is typically more active in transformed cells than in normal counterparts, thus accounting (at least in part) for greater PpIX accumulation in the former^[27,28]^. PpIX levels build up initially in mitochondria, where the penultimate step in heme biosynthesis occurs^[28]^. In addition to sensitizing cytotoxic reactions, ALA-induced PpIX produces a strong red fluorescence under relatively low intensity exciting light. Many oncologists, particularly those treating glioblastoma, have exploited this property for fluorescence-guided resection (FGR), i.e., to clearly demarcate tumor extremities before surgical removal, thereby greatly improving procedural accuracy^[9,29,30]^. Consequently, ALA-induced PpIX has the advantage of serving as a surgical guide on the one hand and a cytotoxic PDT sensitizer on the other hand. The results of a Phase III clinical trial based on this approach were highly promising^[29]^. In this trial, GBM patients treated with ALA, followed by FGR and then PDT, survived twice as long as patients subjected to conventional surgery alone (53 weeks *vs*. 25 weeks).

## ROLE OF NO IN PDT RESISTANCE AND CELL AGGRESSIVENESS: BACKGROUND WORK

The tumor-supporting role of endogenous low flux NO has been recognized for many years, but surprisingly few investigations have dealt with how this NO might affect PDT efficacy. An early study by Gilissen *et al.*^[31]^ using a rat aorta model showed that PDT impairs NO generation by vascular endothelial cells, suggesting that the anti-tumor effects of PDT might be due to vasoconstriction of the tumor microvasculature. In another *in vitro* study, Gupta *et al.*^[32]^ reported that nNOS in epidermoid cancer cells was rapidly upregulated by PDT-like stress and that the resulting NO was cytotoxic, enhancing the effects of PDT *per se*. However, the NO levels attained were not measured, and it appears unlikely that they were sufficient to result in lethal damage. The more likely possibility that upregulated NO was relatively low in concentration and signaled for cytoprotection instead of lethality was not considered^[32]^.

How NO produced by tumor cells or proximal stromal cells might affect PDT efficacy *in vivo* was first investigated about 20 years ago by Henderson *et al.*^[33]^ and Korbelik *et al*.^[34]^, using mouse syngeneic tumor models and Photofrin® as PS. These studies revealed that PDT cure rate could be significantly improved when a general inhibitor of NOS enzymatic activity, e.g., L-NAME or L-NNA, was administered immediately after tumor irradiation^[33,34]^. The extent of improvement correlated with the ability to generate NO, i.e., tumors with the highest outputs responded best, while those with the lowest outputs responded poorly and were much more susceptible to PDT repression^[34]^. More recently, Reeves *et al.*^[35]^, using mouse syngeneic tumors sensitized not with Photofrin®, but ALA-induced PpIX, showed again that endogenous NO exerted a negative effect of PDT efficacy. The findings in each of these studies^[33–35]^ were attributed to NO’s vasodilatory effects acting in opposition to PDT’s recognized tumor-impairing vasoconstrictive effects. It was apparent that measuring NO output of any given tumor might serve as a useful predictor of its PDT vulnerability. This work^[33,34]^ was groundbreaking in identifying NO-mediated resistance to PDT *in vivo*, but it left many important questions unanswered, including: (1) the cellular source(s) of resistance-enhancing NO, e.g., tumor cells themselves, vascular endothelial cells, macrophages, or all of these; (2) which NOS isoform was the major source of resistance NO; (3) whether the NOS in question acted at a basal (constitutive) level or at a stress-induced level; and (4) the underlying mechanism(s) of NO-mediated resistance.

Over the past 20 years, the authors and co-workers have made considerable progress in addressing these questions. Several human cancer cell lines (e.g., breast COH-BR1, MCF-7, and MDA-MB-231 and prostate PC3 and DU145) were sensitized in mitochondria with ALA-induced PpIX and exposed to a moderate dose of broad-band visible light, which activated intrinsic apoptosis in each case^[36–40]^. The extent of cell death (typically 20%−30% at 24 h post-irradiation) increased dramatically when an iNOS competitive inhibitor (1400W or GW274150) or a NO scavenger (cPTIO) was introduced immediately before or after irradiation. From these results, we reasoned that iNOS-generated NO was playing a key role in the acquired resistance to photokilling. This resistance was substantially blunted by shRNA-induced iNOS knockdown prior to cell challenge^[38]^, confirming iNOS involvement in the resistance response. For each of the indicated cell types, iNOS (but not other isoforms, if expressed) underwent a striking upregulation at both the transcript and protein levels after challenge. For example, iNOS was upregulated 8–10-fold in PC3 cells^[39,40]^; thus, the NO it generated would have been much more important in acquired resistance than that from pre-existing (basal level) enzyme. Cells of each type that were able to survive photooxidative stress exhibited a pronounced increase in proliferation, migration, and invasion rates, and these responses were substantially (if not completely) dependent on induced iNOS/NO^[38–40]^.

Using female *SCID* mice engrafted with MDA-MB-231 tumors, Fahey and Girotti^[41]^ showed that the *in vitro* resistance described above^[36–40]^ could be recapitulated at the *in vivo* level. After ALA administration, tumors were irradiated with 633 nm light, using an LED source. These animals exhibited a significant slowdown in tumor growth compared with light-only controls. However, multiple doses of 1400W or GW274150 slowed growth much more, indicating iNOS/NO was promoting resistance just as observed with MDAMB-231 cells *in vitro*^[41]^. On the other hand, the iNOS inhibitors had no significant effect on light-only controls, suggesting that pre-existing iNOS/NO made little, if any, contribution to any basal resistance^[41]^. This agrees with our conclusions about *in vitro* resistance (see above). Western blot analysis of post-PDT tumor samples revealed a striking 5-fold upregulation of iNOS, along with a 1400W-inhibitable increase in NO-derived nitrite^[41]^. This was the first reported evidence for iNOS/NO-imposed resistance to tumor repression by PDT in a human xenograft model.

## GLIOBLASTOMA CELLS: ROLE OF NO IN PDT RESISTANCE AND ACQUIRED AGGRESSIVENESS

**Figure 3.**
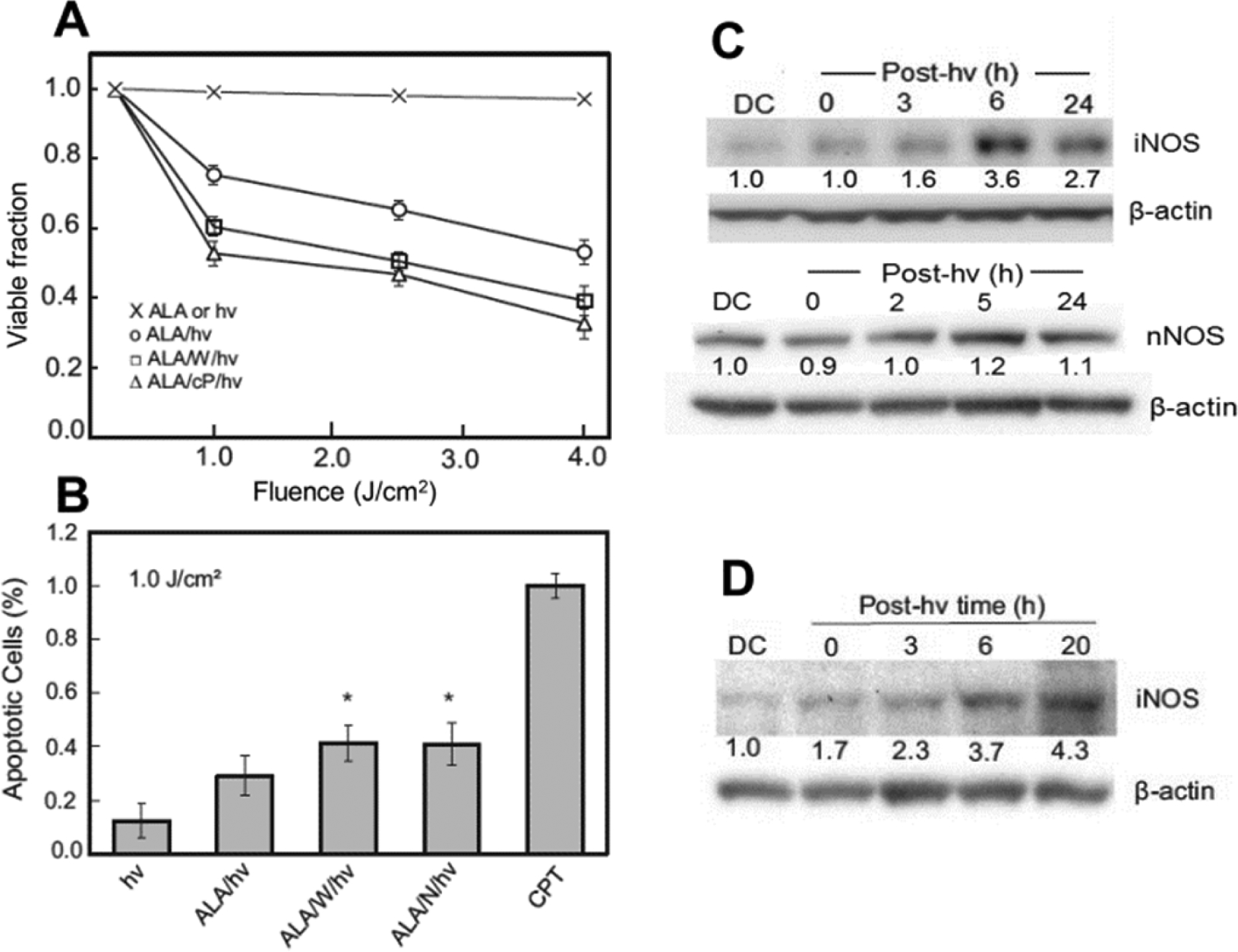
Photokilling of glioblastoma cells: inhibitory effects of nitric oxide from stress-upregulated inducible NO synthase (iNOS). (A) aminolevulinic acid (ALA)-treated U87 cells were irradiated with broad-band visible light in the absence (○ ) vs. presence of 1400W ( □ ) or cPTIO (Δ). A light-only or ALA-only control was run alongside (×); (B) U87 apoptosis after ALA/light treatment: stimulation by 1400W (W) or L-NAME (N). Values are relative to a camptothecin (CPT) standard; (C) immunoblot of iNOS and nNOS in photostressed U87 cells; (D) immunoblot of iNOS in photostressed U251 cells. (D,C): DC represents ALA-only dark control. Numbers below bands indicate NOS band intensity relative to β-actin and normalized to DC^[42]^

Similar to the other cancer cell lines mentioned, human glioblastoma U87 cells sensitized with ALA-induced PpIX exhibited a loss of viability after irradiation^[42]^ and this increased progressively with increasing light fluence [Figure 3A]. Controls treated with ALA alone or light alone remained completely viable, indicating that sensitized photodynamic action was necessary for cytotoxicity.

When sensitized cells were irradiated in the presence of 1400W or cPTIO, there was a large increase in viability loss [Figure 3A], thereby implicating iNOS/NO in photokilling resistance, as had also been concluded for breast and prostate cancer cells^[37–40]^. ALA/light-induced cytotoxicity was also assessed in terms of apoptosis, the extent of which was significantly greater when 1400W or L-NAME was present [Figure 3B]. After an ALA/light challenge, surviving (still attached) U87 cells displayed a progressive increase in iNOS protein during post-hν incubation, the level at 6 h being nearly four times that of a dark control [Figure 3C]. In contrast, nNOS, which was abundantly expressed by these cells, showed no significant increase over its basal level. Thus, nNOS appears not to have made any significant contribution to the acquired stress resistance in these cells. Another established glioblastoma line, U251 cells, exhibited similar iNOS/NO-mediated resistance to an ALA/light challenge^[42]^; this was accompanied by a steady upregulation of iNOS protein, which reached ~4 times the control level 20 h after irradiation [Figure 3D]. Elevated iNOS expression in U87 cells was accompanied by a large increase in NO output, as detected with the fluorescence probe DAF-2DA. The fluorescence signal at 4 h after irradiation was ~3 times that of an ALA-only control and was strongly inhibited by 1400W^[42]^, as expected for iNOS-generated NO.

**Figure 4.**
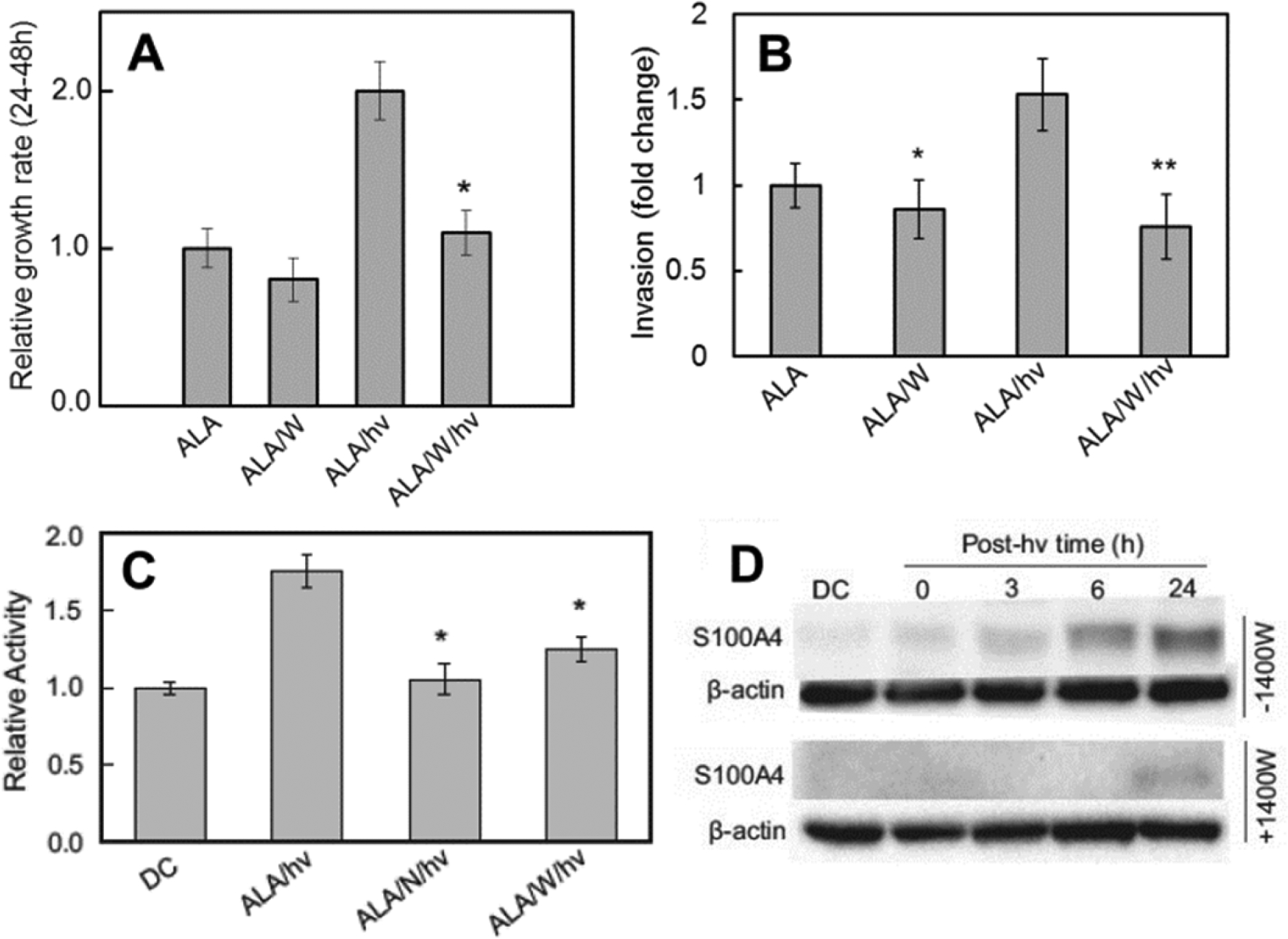
Increased proliferation rate, invasion rate, MMP-9 activity, and S100A4 expression in photostressed U87 cells: inducible nitric oxide (NO) synthase/NO-dependency. A: 1400W-inhibited proliferation of surviving cells from 24–48 after aminolevulinic acid (ALA)/light treatment. Also shown: insignificant effect of 1400W on ALA-only control; B: 1400W-inhibited invasion of surviving cells 24 h after ALA/light ytratment; **P < 0.01 vs. ALA/light; C: 1400W- and L-NAME-inhibitable MMP-9 activation. Extracellular enzyme after ALA/light was concentrated and analyzed for activity by in-gel zymography. *P < 0.01 vs. ALA/hν; D: 1400W-inhibitable S100A4 upregulation after an ALA/light challenge^[42]^

Tumor cells often respond to stress conditions by becoming more aggressive in terms of proliferation and mobility^[43]^. Thus, it was important to learn whether PDT-challenged glioblastoma cells could exploit iNOS/NO not only for resistance to photokilling, but also greater proliferative and migratory aggressiveness. As shown in Figure 4A, U87 cells that remained viable 24 h after ALA/light treatment exhibited a strong growth spurt (~2-fold) compared with ALA-only controls^[42]^. This spurt was nearly nullified by 1400W or cPTIO (not shown) but each showed only a small (insignificant) inhibitory effect on a dark control (ALA-only). This demonstrates the strong influence that upregulated NO had on cell growth compared with basal NO. Two other manifestations of hyper-aggressiveness were also observed after an ALA/light challenge: (1) accelerated migration into a cell-depleted zone; and (2) accelerated invasion through an interface resembling the extracellular matrix-(ECM). Using a gap-closure or “wound-healing” assay, Fahey *et al.*^[42]^ found that ALA/light-stressed U87 cells consistently migrated more rapidly than dark controls and in 1400W-inhibitable fashion. One experiment revealed a 45% greater rate for photostressed cells over a 24 h post-irradiation period. 1400W also slowed control cell migration, but to a much smaller extent than stressed counterparts, again demonstrating the dominance of stress-upregulated iNOS/NO. To assess invasiveness, Fahey *et al.*^[42]^ used a 96-place trans-well device with Matrigel-infused filters. The ability of U87 cell to traverse from serum-free upper wells to serum-containing lower wells was determined over a 24-h post-irradiation period. As shown in Figure 4B, photostressed cells displayed a remarkable 50% increase in invasion rate relative to controls. As anticipated, this increase was strongly blunted by 1400W, whereas the latter had only a small (barely significant) effect on the ALA-containing dark controls. Thus, greater invasiveness, similar to migration, was strongly dependent on iNOS/NO. Matrix metalloproteinases (MMPs) such as zinc-containing MMP-9 catalyze the degradation of collagen and other ECM components, and thus play a key role in cancer cell invasiveness and metastasis^[44]^. MMP-9 is known to promote innate migration/invasion of glioma cells^[45,46]^. Therefore, it was of interest to assess its possible involvement in PDT-aggravated U87 aggressiveness. Fahey *et al.*^[42]^ found that total immunodetectable MMP-9 underwent a slow upregulation after an ALA/light challenge, increasing to ~150% of its control level after 24 h. When the activity of externalized MMP-9 was measured by gelatin zymography, it was found to be at least 80% greater than that of control enzyme [Figure 4C]. Strong inhibition by L-NAME and 1400W demonstrated that this activation (e.g., the increased invasiveness) was substantially iNOS/NO-dependent [Figure 4C]. Based on “cysteine switch” domain considerations^[47]^, activation may have occurred via NO binding to a Zn(II) ion in pro-MMP-9, leading to release of an activity-repressing peptide segment. Of added importance was the observation that a tissue inhibitor of metalloproteinases (TIMP-1) was progressively downregulated by photostress, and in a 1400W-inhibitable fashion, thus revealing an intricate NO-controlled relationship between MMP-9 and TIMP-1. Two other proteins known to play important roles in tumor progression exhibited 1400W-inhibitable upregulation in photostressed U87 cells: Survivin and S100A4^[42]^. Immunoblot results obtained with metastasis-promoting S100A4 were particularly striking, since it was barely detectable initially, but underwent a remarkably strong upregulation after ALA/light treatment, which was effectively abrogated by 1400W [Figure 4D]. Thus, photostress-induced NO signaled for altered status of several key tumor promoting proteins: MMP-9 (activation), TIMP-1 (downregulation), Survivin (upregulation), and S100A4 (upregulation).

## UNDERLYING MECHANISMS OF INOS/NO PRO-SURVIVAL EFFECTS

**Figure 5.**
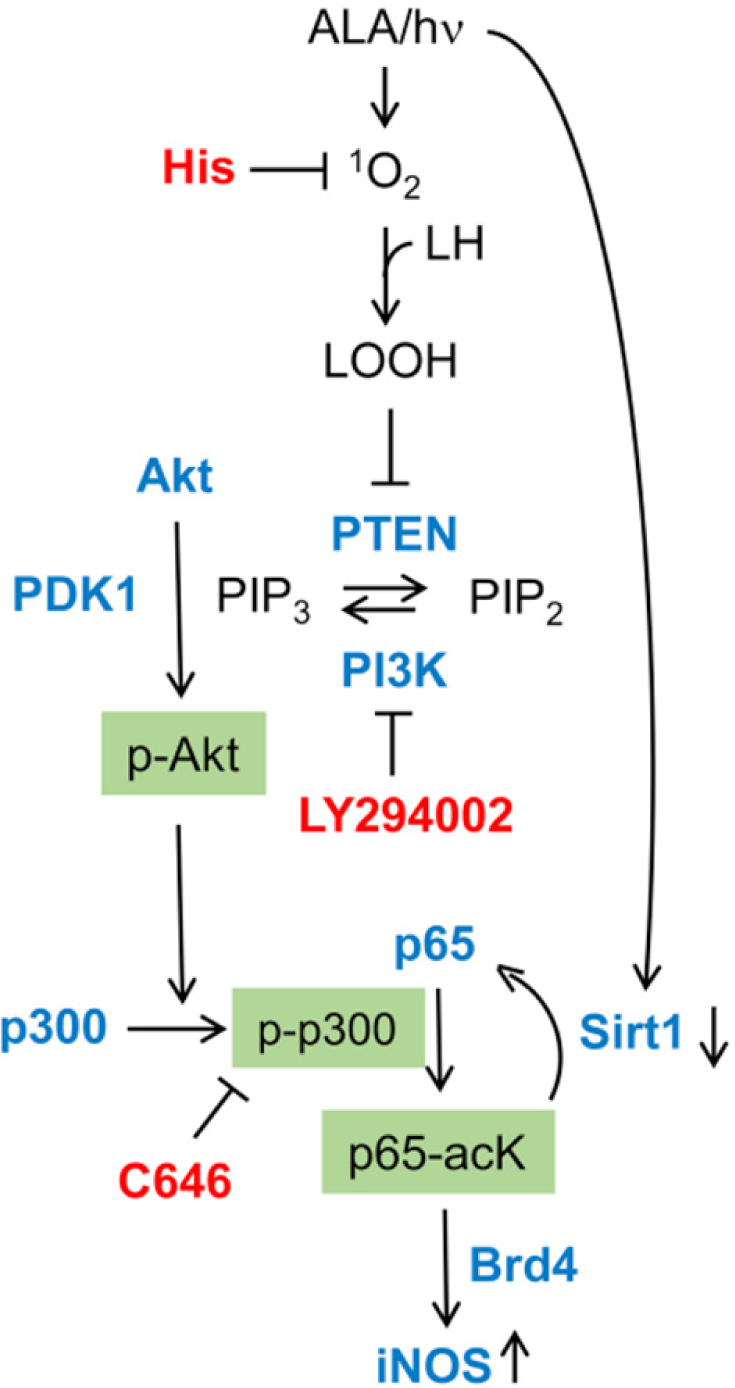
Upstream signaling events elicited by photodynamic therapy (Ala/light) leading ultimately to inducible nitric oxide synthase (iNOS) transcriptional upregulation. Key effector proteins (Akt, PI3K, p300, p65, Sirt1) are shown along with key inhibitors (His, LY294002, C646) and their targets^[48]^

For mechanistic understanding, most efforts to date have focused on how iNOS/NO is upregulated rather than how NO signals for greater cell resistance or aggressiveness, although some progress has been made in the latter category. Working with U87 and U251 cells, Fahey and Girotti^[48]^ found that transcription factor NF-κB played a seminal role in post-ALA/light iNOS induction leading to greater cell migration and invasion. Consistent with this, NF-κB subunit p65/Rel A of photostressed U87 cells translocated from cytosol to nucleus, where it participated in iNOS transcription^[48]^. Based on non-glioma studies by Huang *et al.*^[49]^, it was postulated that specific lysine acetylation on p65 is necessary for stimulating transcriptional activity. Fahey and Girotti^[48]^ verified this by showing that acetylation of lysine-310 (p65-acK310) is substantially upregulated in photostressed U87 cells. Investigation of upstream signaling events revealed that p65-acK310 formation was dependent on phosphorylation-activation of PI3K, followed by Akt, and thence acetyltransferase p300. These sequential activations were supported (at least in part) by inactivation of tumor-suppressor PTEN (PIP_3_ phosphatase) via intramolecular -S-S- bond formation^[48]^. The rise in acK310 level after photostress was suppressed by an inhibitor of activated p300, confirming that the latter had catalyzed acetylation of p65-K310^[48]^. Accompanying these effects was a strong upregulation of Brd4 (bromodomain protein type-4), an epigenetic “reader” and transcriptional co-activator for several stress-responding genes, including iNOS^[50–52]^. Moreover, pull-down analyses revealed a striking stress-enhanced interaction of p300 and p65, thus facilitating acetylation of the latter for transcriptional activity^[48]^. Observing Brd4-regulated iNOS transcription in these studies was entirely novel with regard to basal or therapy-stimulated cancer cell aggressiveness. Another remarkable finding in this work was that Sirtuin-1 (Sirt1), a Class-III deacetylase that modulates gene expression via removal of acetyl groups on histones and certain transcription factors^[53,54]^, was strongly downregulated in photostressed U87 cells. In contrast, the protein level of another Class-III deacetylase, Sirt2, was unaffected, suggesting specificity for Sirt1 in the stress response. When considered as a whole, the above findings reveal a remarkably well-orchestrated and cooperative upstream signaling network that leads ultimately to upregulation of pro-survival/expansion iNOS/NO [Figure 5]. Such signaling could also play a role in upregulation/activation of other stress-responsive proteins, e.g., MMP-9, COX-2, and S100A4^[42]^. In considering pro-survival mechanisms of photostressed tumor cells, it is important to bear in mind that PDT *in vivo* typically results in a boost in anti-tumor immunity which can enhance the overall efficacy of this therapy^[26]^. However, it is now well established that iNOS-generated NO in myeloid-derived suppressor cells (MDSCs) can impair anti-tumor immunity by inactivating cytotoxic T-lymphocytes^[55]^. This could be another mechanism by which endogenous NO might compromise anti-glioblastoma PDT, but whether it actually applies for this particular malignancy remains to be determined.

## PRO-TUMOR BYSTANDER EFFECTS OF PDT-UPREGULATED NO

A new facet of NO-mediated antagonism to PDT was discovered about 4 years ago, *viz*. increased aggressiveness cells that had not been directly affected by photodynamic action, i.e., bystander cells. Since most established tumors, including glioblastomas, have defective vascular systems^[1,2]^, not all cells will be uniformly supplied with an administered PS or pro-PS such as ALA. Furthermore, during subsequent irradiation, some cells would inevitably be less exposed than others due complex factors such as limitations of the light field, light scattering, etc. One can postulate that PDT-targeted cells can send signals to nonor minimally-targeted counterparts (bystanders) which stimulate growth and mobility of the latter. Such effects have been well documented for ionizing radiation (e.g., X-rays and α-particles), for which various diffusible mediators have been described, including cytokines, H_2_O_2_, and NO. Of special interest here are studies by Matsumoto *et al.*^[56,57]^ showing that NO from upregulated iNOS in X-ray-targeted glioblastoma cells imposed a significant radioresistance in non-targeted bystander cells. To assess whether such effects might be applicable to ALA-based PDT, Bazak *et al.*^[58]^ developed a novel approach in which impermeable silicone-rimmed rings were used to separate targeted cells (ALA/light-treated, outside rings) from non-targeted bystanders (light-only, inside rings) on a large culture dish. After some post-hν interval (e.g., 2 h), rings were removed to allow diffusion of small molecules from targeted cells to bystanders. Responses in both compartments were assessed during subsequent dark incubation, e.g., changes in iNOS/NO levels and in growth and proliferation rates. Prototype experiments with prostate PC3 cells revealed not only the expected iNOS/NO upregulation and growth/migration spurt in surviving targeted cells, but the same responses in non-stressed bystander cells as well^[58]^. Strong mitigation of the latter responses by 1400W, cPTIO, or iNOS knockdown in target cells indicated that NO from the stressed targeted cells was primarily responsible. In addition to iNOS, several other pro-tumor effectors were upregulated in glioblastoma bystanders, including Akt, ERK1/2, and COX-2^[58]^. Bazak *et al.*^[59]^ observed similar bystander effects with glioblastoma U87 cells and compared them with the effects obtained with prostate PC3, breast MDAMB-231, and melanoma BLM cells, using ALA/light conditions that resulted in uniform kill for all four types (~25%). Under these conditions, bystander proliferation and migration rates increased in proportion to the extent of iNOS upregulation in surviving targeted cells according to the following order: PC3 > MDA-MB-231 > U87 > BLM^[59]^. These findings, along with the non-effects of conditioned media from targeted cells, confirmed that continuously generated NO by upregulated target cell iNOS was responsible for stimulating bystander aggressiveness. This evidence suggests that a type of relay process is set in motion during a photodynamic challenge whereby NO overproduced by targeted U87 cells, for example, diffuses to naive bystanders and induces iNOS/NO there, thus beginning a NO “feed-forward” process that propagates through the bystander population. Recognizing this was possible when the above-described means of distinguishing between bystander and targeted cells became available. If occurring in an actual tumor after ALA-PDT, e.g., a glioblastoma, NO-stimulated bystander aggressiveness might result in more rapid tumor growth and metastatic dissemination. As discussed below, these negative effects of surviving targeted cells could be mitigated by PDT adjuvants that either inhibit iNOS activity or iNOS transcription.

## PHARMACOLOGICAL APPROACHES FOR SUPPRESSING ANTI-PDT EFFECTS OF NO

As mentioned throughout this review, recognition of iNOS/NO signaling for a survival, proliferative, and migratory advantage in tumor cells is often based on the mitigating effects of specific iNOS activity inhibitors (1400W and GW274150) or a NO scavenger (cPTIO). For glioblastoma cells *in vitro*, such agents have been indispensable for identifying pro-growth/migration signaling of endogenous iNOS/NO after an ALA/light challenge^[42,48]^. Might such effects be realized at the clinical level when tumor-repressing PDT is used? Although this has not been attempted yet, there is good reason to believe that certain iNOS inhibitors would improve clinical outcomes when used in conjunction with PDT. Reflecting favorably on this is the fact that two such inhibitors, L-NIL and GW274150, have already been tested in clinical trials, although these were unrelated to cancer or PDT^[60,61]^. Both were tested for ameliorating asthmatic inflammation, and neither one elicited any unfavorable side effects. As indicated above, GW274150 significantly improved PDT efficacy in a human tumor xenograft model^[41]^, suggesting that this inhibitor would be a good test candidate as a PDT adjuvant.

As already mentioned, iNOS transcription in glioblastoma cells is regulated by NF-κB subunit p65, which becomes activated via p300-catalyzed acetylation of lysine-310. Brd4 protein, which contains bromodomain and extra-terminal (BET) domains, acts as an iNOS transcriptional co-activator by binding to p65-acK310^[48]^. JQ1, a synthetic inhibitor of Brd4 and other BET proteins, acts by binding to BET domains, thereby preventing interaction with a acK groups on transcription factors (e.g., p65-acK310) or on histones^[62]^. When tested on ALA/light-treated U87 cells, BET inhibitor JQ1 at a minimally cytotoxic level (300 nmol/L): (1) acted synergistically with PDT in killing cells; (2) strongly inhibited Brd4’s ability to interact with p65-acK320 after PDT; (3) nearly abolished iNOS upregulation after PDT; and (4) prevented PDT-surviving cells from becoming more aggressive in proliferation and invasion^[63]^. JQ1 inhibited these negative responses to PDT at ~100 times lower concentration than 1400W, making it a highly promising PDT adjuvant, particularly since it has already been used successfully with other anti-cancer therapies. In the case of glioblastoma, for example, JQ1 has synergized with temozolomide, both *in vitro* and *in vivo*^[64]^. Transcriptional upregulation of pro-survival/expansion iNOS under stress-inducing therapies such as PDT may occur more often than presently recognized, thus emphasizing the need for powerful inhibitors like JQ1 as therapeutic adjuvants.

## SUMMARY AND PERSPECTIVES

PDT for solid malignancies such as glioblastomas has many advantages over other treatment options, tumor site-specificity being a major advantage. However, as with other anti-tumor modalities, e.g., radiotherapy and chemotherapy, PDT is often confronted with pre-existing or treatment-induced resistance, which can reduce overall efficacy. Exacerbating this is the fact that tumor cells surviving photodynamic stress inevitably acquire a more aggressive phenotype in terms of proliferation and migration/invasion. Endogenous iNOS/NO has been shown to play major role in each of these negative responses, particularly iNOS/NO that is upregulated by PDT stress. Most therapy-based studies to date, including those on glioblastoma, have neither considered the possibility of iNOS upregulation during treatment nor that the resulting NO might be more important in enhancing resistance and aggressiveness than NO from pre-existing enzyme. Given that PDT is now frequently used for repressing glioblastoma, it is imperative that the negative effects of iNOS/NO (e.g., the more invasive potential of surviving cells) be eliminated, or at least mitigated, by reliable pharmacologic interventions. Existing iNOS activity inhibitors, although highly effective in cell and animal models, lack tumor selectivity and could be problematic for off-target effects. Alternatively, suppression of iNOS transcription with a BET inhibitor such as JQ1 has great promise, especially since several of these agents are proving effective in clinical trials for a variety of malignancies. In addition to iNOS, BET inhibitors should impair transcription of other pro-tumor genes, e.g., *Bcl-xL*, Survivin, and MMP-9^[63]^, thus resulting in an even greater suppression of post-PDT negative effects. Thus, PDT outcomes for glioblastoma should be greatly improved with the introduction of BET inhibitors as adjuvants.
